# Epidemiological and clinical characteristics of patients with human monkeypox infection in Zhejiang Province, China, 2023

**DOI:** 10.3389/fpubh.2025.1528679

**Published:** 2025-06-02

**Authors:** Xuguang Shi, Ying Liu, Rong Zhang, Jiangping Ren, Song Guo, Zhen Wang, Jimin Sun

**Affiliations:** Zhejiang Provincial Center for Disease Control and Prevention, Hangzhou, China

**Keywords:** monkeypox, clinical characteristics, epidemiology, rash, fever

## Abstract

**Background:**

Monkeypox (Mpox), a zoonotic disease caused by the monkeypox virus (MPXV), is endemic in parts of Central and West Africa. Since 2022, an outbreak of the Mpox infection emerged in several non-endemic countries, posing a potential threat to human health. The first case in Zhejiang Province was confirmed in Hangzhou City on June 15, 2023. The objective of this study is to delineate the epidemiological and clinical characteristics of monkeypox cases in Zhejiang Province in 2023, thereby providing a foundation for prevention and control measures.

**Methods:**

Data on all the confirmed monkeypox cases were collected to describe the epidemiological and clinical characteristics of monkeypox cases in Zhejiang Province in 2023. The spatial distribution of monkeypox cases was explored by means of ArcGIS software.

**Results:**

In 2023, a total of 182 cases of monkeypox were reported in Zhejiang Province, with no deaths. All infections represented autochthonous transmission and reported in 40 counties (districts) of 9 cities, except for Zhoushan and Lishui. All cases were male, with a median age of 29 years. Among them, 177 cases were men who have sexed with men (MSM), including 26 bisexual individuals. 43.96% of the cases had been previously infected with HIV. The five common clinical features of the monkeypox cases were rash or skin lesions, fever, itching, ache and lymphadenopathy. The most common sites of the lesions were the genitals, limbs, torso, face, and mouth.

**Conclusion:**

The monkeypox epidemic is in a state of transmission and prevalence in some areas of Zhejiang Province. Therefore, it is necessary to maintain very close surveillance. A significant majority of laboratory-confirmed mpox cases were identified among men who have sex with men (MSM). Tailored interventions, including ‌mpox-specific symptom monitoring‌, health education, must be urgently implemented for MSM populations in high-transmission settings.

## Introduction

Monkeypox, a zoonotic disease caused by monkeypox virus, was initially detected in 1958 among monkeys, and the first confirmed human case of monkeypox was reported in the Democratic Republic of the Congo in 1970 (1, 2). Sporadic cases of monkeypox have been mainly reported in endemic regions of parts of Central and Western African countries from 1970 to 2017, typically resulting from exposure to wild animals, especially rodent species (3, 4). In 2003, the first outbreak of human monkeypox outside Africa occurred in the United States, resulting in 47 cases. The cause was exposure to infected animals that had co—lived with infected small mammals imported from Ghana. There was no clear evidence of human—to—human transmission (5). From 2018 to 2021, sporadic cases were detected in countries outside Africa, such as the United Kingdom, Singapore and Israel (6–8).

Since May 2022, monkeypox cases have been reported in a multitude of non-epidemic regions such as the United Kingdom, Spain, Germany, Portugal, France, Canada, United States, Japan, South Korea, and Singapore so on (9). As of 30 April 2024, a total of 97,208 laboratory confirmed cases and 609 probable cases, including 186 deaths, have been reported to WHO (10).

The virus was mainly transmitted through the following routes: physical exposure to infectious skin lesions (particularly those containing viral-laden exudates); respiratory droplet transmission; contact with items contaminated with the MPXV‌. During the global outbreak in 2022, a significant increase in transmission efficiency was observed among MSM. Sexual contact transmission constituted the predominant mode of spread during this outbreak, warranting particular attention.

Previous study showed that skin lesions, fever, and lymphadenopathy were the most common symptoms. Skin lesions can appear on various parts of the body, including the face, limbs, hands, trunk, and more (11). However, in the current outbreak, lesions in the anogenital area are the most frequently and earliest observed skin manifestations (12).

Epidemic spatio-temporal maps can be constructed to analyze the spatial clustering of cases, which enables rapid positioning of high-risk areas, optimization of resource allocation, assessment of risk factors, and significantly improves the scientificity and precision of public health emergency responses.

Due to the entry quarantine policy implemented during the COVID—19 prevention and control period, the detection time of imported monkeypox cases in China was delayed compared with other countries. In September 2022, the first imported monkeypox case was confirmed in Chongqing during the quarantine of incoming travelers (13). No secondary infections were found among the close contacts of this case. Subsequently, on May 31, 2023, Beijing reported the first locally—transmitted monkeypox case in Mainland China (14), marking the entry of the monkeypox epidemic into the stage of local transmission. Since then, the epidemic has shown a rapid spread trend. By the end of 2023, a total of 1717 monkeypox cases had been reported in 18 provincial—level administrative regions in Mainland China, indicating a wide geographical distribution. On June 15, 2023, the first confirmed monkeypox case was reported in Hangzhou, Zhejiang Province. Epidemiological investigations show that the MSM (men who have sex with men) population is currently the main high—risk group for MPXV infection. Based on the current epidemic situation, this study aims to systematically understand the epidemiological distribution characteristics of the monkeypox epidemic in this region, accurately identify key prevention and control areas, and deeply analyze the epidemiological characteristics and early —clinical manifestations of the cases. This will provide a scientific basis for establishing an early identification and diagnosis mechanism for monkeypox cases and formulating precise prevention and control strategies that are in line with the actual situation of this region.

## Materials and methods

### Data source

The data of monkeypox cases in Zhejiang Province was obtained from the China Information System for Disease Control and Prevention (CISDCP). The information of the cases was collected including age, gender, date of onset and date of reporting, place of residence, place of reporting, occupation and so on. The retrieval criteria were as follows: The date of onset is between January 1, 2023, and December 31, 2023, and the current place of residence is Zhejiang Province.

Epidemiological investigations are conducted by public health professionals following the successful completion of standardized training programs and competency-based evaluations, in accordance with national disease surveillance protocols. The case investigation form designed by the Chinese Center for Disease Control and Prevention was used to collect information such as the basic details of the cases, human immunodeficiency virus (HIV) infection status, clinical manifestations, medical treatment, suspected sources of infection, close contacts and so on.

### Case definition

The diagnosis and determination of monkeypox were based on the “Technical Guidelines for Monkeypox Prevention and Control (2022 Edition)” (15). A confirmed case of monkeypox was defined by the presence of skin or mucosal lesions and at least one human sample testing positive for the MPXV.

Specimens from the site of skin lesions (swabs from the surface of the lesions, pox vesicle fluid) and respiratory tract specimens (oropharyngeal swabs) were collected. The testing of specimens was implemented by disease prevention and control institutions. The extraction of MPXV nucleic acid was accomplished using the Fluorescence quantitative real-time PCR method.

### Statistical analysis

Descriptive statistics were carried out to illustrate the general characteristics of the monkeypox cases in Zhejiang Province in 2023. Using the vector map of the administrative divisions of Zhejiang Province as the base map, and by using the ArcGIS 10.7 software for spatial mapping, the number of cases reported in each county and district of Zhejiang Province is presented, which can intuitively show the distribution of monkeypox cases in different regions of Zhejiang Province. For continuous variables, medians together with either interquartile ranges (IQRs) or ranges were presented. Categorical variables were expressed in terms of frequency and percentage. The normality of data distribution was assessed using R software (significance level *α* = 0.05).

## Results

On June 15, 2023, the first local case of monkeypox was confirmed in Gongshu District, Hangzhou City of Zhejiang Province. The patient had neither a travel history to outbreak-epidemic countries nor close contact with monkeypox patients. Epidemiological investigation demonstrated that the source of infection for the patient was uncertain, and all same-sex partners or other close contacts tested negative.

**Figure 1:**
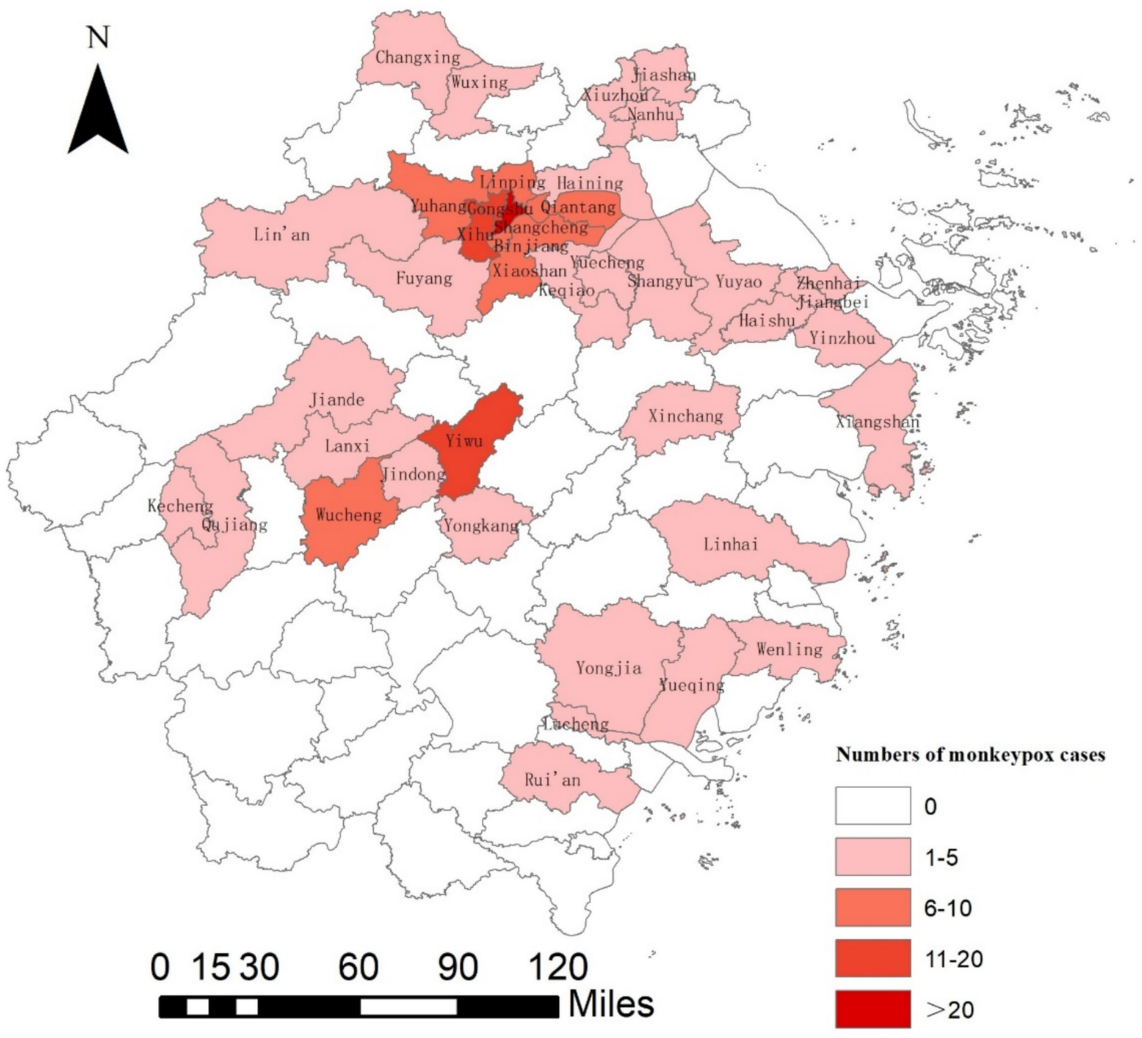
The spatial distribution of confirmed monkeypox cases in Zhejiang Province, 2023.

In 2023, a total of 182 cases of monkeypox were reported in Zhejiang Province, with no death case (an incidence rate of 0.28 per 100,000). Among these, 179 cases had no history of travel to affected countries within 21 days before the onset of the illness, and the other three cases had no suspicious contact history during overseas travel. All subjects were locally acquired and showed no exposure to blood, bodily fluids, or excretions from MPXV-infected animals, including rodents and non-human primates. Monkeypox cases were reported in 40 counties (districts) of nine cities in Zhejiang Province except for Zhoushan and Lishui (Figure 1), with the highest number of reported cases in Hangzhou at 119 (accounting for 65.38% of all cases in the province), followed by Jinhua with 25 cases, Ningbo with 11 cases, Wenzhou with 7 cases, Jiaxing with 6 cases, Shaoxing and Taizhou with 5 cases each, and Quzhou and Huzhou with 2 cases each. The top five regions with reported cases were Shangcheng District (36 cases), Gongshu District (20 cases), Xihu District (14 cases), Yiwu City (12 cases), and Yuhang District (10 cases).

The demographic information and the human immunodeficiency virus (HIV) infection status of the confirmed monkeypox cases were presented in Table 1. All the confirmed cases were male, and the median age was 29.00 years (IQR: 26.00–34.75, range: 19.00–52.00, *p* < 0.05). The majority were unmarried (83.5%), and males in the age range of 20–39 years old constituted the main population, accounting for 89.01% of the reported cases. Among them, 177 cases (97.25%) were MSM, including 26 bisexual individuals. The epidemic has not yet affected the female spouses of bisexual couples, and the test results were negative. 123 traceable close contacts, excluding sexual contact, had not been infected. The predominant occupational distribution comprised ‌commercial service workers‌ (*n* = 74; 40.7%), followed by ‌manual laborers‌ (*n* = 36; 19.8%) and ‌homemakers/unemployed individuals‌ (*n* = 20; 11.0%). Two cases involving college students were reported on campuses in Quzhou and Jiaxing.

**Table 1:** The demographic information and HIV infection status of the confirmed monkeypox cases in Zhejiang Province in 2023.

Characteristics	Number of patients
Sex	
Male	182(100%)
Female	0 (0%)
Age groups, median (IQR) age (years)	
<20	1 (0.55%)
20–29	92 (50.55%)
30–39	70 (38.46%)
40–49	16 (8.79%)
≥50	3 (1.65%)
Marital status	
Married	9 (4.94%)
Unmarried	149 (81.87%)
Divorced	24 (13.19%)
Occupation	
Business services	74 (40.66%)
Workers	36 (19.78%)
Household chores and unemployed	20 (10.99%)
Cadres and staff	17 (9.34%)
Farmer	9 (4.95%)
Catering and food industry	2 (1.10%)
Medical staff	1 (0.55%)
Student	2 (1.10%)
Other	10 (5.49%)
Missing	11 (6.04%)
HIV-positive	80 (43.96%)
Travel abroad 21 days before onset	3 (1.65%)
Smallpox vaccination	
Yes	3 (1.65%)
No	93 (51.10%)
No information provided	86 (47.25%)

176 cases were discovered through active medical consultations (96.27%), except for 6 cases discovered through close contact screening. We documented 6 clustered events involving 11 cases; the largest number of previously recorded events was 3. All close contacts except for sexual contacts were negative.

The time interval from onset to diagnosis of 176 active cases ranged from 1 day to 24 days, with a median time of 7 days (IQR: 5–10, range: 1–24, *p* < 0.05), and those over 10 days accounted for 24.43%. The median time from the initial visit to diagnosis was 3 days (IQR: 1–5.25, range: 1–22, *p* < 0.05), and 25% of the cases had an interval of more than 5 days.

The clinical characteristics are presented in Table 2. A total of 181 cases of MPXV infection (181/182) were symptomatic, and the most prevalent self-reported symptoms were rash/skin lesions (180 cases, 98.9%) and fever (120 cases, 65.93%)‌. Among 182 cases, 53.85% (98 cases) presented with lesions as the initial symptom, while 30.22% (55 cases) first developed fever‌. Fever and lesions appeared on the same day in ten patients.

**Table 2:** Clinical characteristics of the persons with monkeypox in Zhejiang Province, 2023.

clinical characteristics	Number of patients
Rash or skin lesions anywhere on the body	180 (98.90%)
Genitals	91 (50%)
Limbs	75 (41.21%)
Torso	52 (28.57%)
Face	47 (25.82%)
Mouth	44 (24.18%)
Perianal	40 (21.98%)
Neck	4 (2.20%)
Throat	3 (1.65%)
Other	3 (1.65)
Fever	120 (65.93%)
37.3°C or higher	24/120 (20%)
38°C or higher	50/120 (41.67%)
39°C or higher	24/120 (20%)
Not measured or provided	22/120 (18.33%)
Lymphadenopathy	34 (18.68%)
Itching (pruritis)	69 (37.91%)
Headache	28 (15.38%)
Muscle aches	24 (13.19%)
pain at the site of the rash	57 (31.32%)
Fatigue or malaise	5 (2.75%)
Diarrhea	4 (2.20%)
Sore throat (pharyngitis)	3 (1.65%)
Runny nose (rhinitis)	2 (1.10%)
Chills	1 (0.55%)
Sweats	1 (0.55%)
Cough	1 (0.55%)
Constipation	1 (0.55%)
Pus or blood in stool	1 (0.55%)

The most common sites of lesions were the genitals (91, 50%), limbs (75, 41.21%), torso (52, 28.57%), face (47, 25.82%), mouth (44, 24.18%) and perianal (40, 21.98%). The intensity of fever caused by the MPXV can range from mild to severe. Among the 120 cases with fever that had temperature measurement, 16 (69.6%) had a temperature of 38.0°C or higher, and 7 (30.4%) had a temperature of 39.0°C or higher (Table 2).

Other common clinical symptoms or signs included itching (69, 37.91%), lymphadenopathy (34, 18.68%), pain at the site of the rash (57, 31.32%), fatigue or malaise (5, 2.75%), headache (28, 15.38%), muscle aches (24, 13.19%), diarrhea (4, 2.20%), and sore throat (3, 1.65). Lymphadenopathy were found in 34 cases (18.68%), and the common region of lymphadenopathy was the inguinal (30, 88.24%).

Of the 182 cases analyzed, 80 (43.96%) were identified as HIV-seropositive.8 cases were born prior to 1980, three of whom self-reported having received smallpox vaccination.

## Discussion

This study was the first research to describe the clinical and epidemiological characteristics of monkeypox cases in Zhejiang Province. We described the epidemiological and clinical characteristics of 182 laboratory-confirmed cases of Monkeypox in nine cities of Zhejiang Province. We observed that monkeypox is currently in a relatively low-prevalence state in Zhejiang Province (incidence rate of 0.28 per 100,000). The reasons for the high incidence in the five areas of Shangcheng, Gongshu, Xihu, Yuhang and Yiwu may be related to the population size, density and medical conditions. According to the public data on the website of the Ministry of Civil Affairs of China, the permanent population of these areas all exceeds 750,000, and the population density of the first four counties and districts is higher than 1,000 people per square kilometer. A large population base and high population density have led to a relatively large potential MSM population in these areas, which may be one of the epidemiological factors contributing to the high incidence of monkeypox. In addition, as a regional medical center, Hangzhou has concentrated a number of high—quality medical institutions and has strong disease screening and diagnosis capabilities.

Consistent with previous studies (16, 17), our study found that MSM was the predominant risk group for mpox (97.25%) in Zhejiang Province, with the MPXV mainly being transmitted among MSM through same-sex sexual contact. The majority of infections lacked an identifiable source, suggesting transmission dynamics characterized by covertness and diverse transmission routes. The infection situation of the MPXV among the MSM population is not optimistic, and special attention should be given to this group during the clinical diagnosis and treatment of monkeypox cases.

In our study, a significant proportion (43.96%) of monkeypox cases were also reported to be associated with HIV infection, similar to previous reports (18). Screening for the MPXV needs to be strengthened in the HIV population.

It is considered that MPXV has a limited ability to spread in human populations (19, 20). No secondary cases were found among the close contacts of confirmed patients other than those through sexual contact. Although both domestic and foreign literature reports suggest that family members may transmit the MPXV through close contact (21, 22), such transmission has not been observed in the cohort of this study. This finding indicates that, in the context of the current epidemic, transmission through intra—family contact may not yet be the main route of MPXV spread in Zhejiang Province. However, considering the potential risks of virus transmission, it is still recommended to implement necessary medical observation and preventive measures for close contacts among family members. The predominance of commercial service workers in the occupational distribution of study subjects may primarily stem from two factors: this demographic is predominantly composed of young and middle-aged adults who generally lack smallpox vaccine-induced immunity, while their frequent social interactions—particularly potential sexual contact transmission pathways—could amplify transmission chains.

Literature reports suggest that smallpox vaccination has an 85% protective effect against monkeypox (23–25). A recent study of 528 cases reported that among the individuals infected with the MPXV, only 9% of them have received the smallpox vaccine (26). China has discontinued smallpox vaccination since 1980. As the majority of cases in this study were born after 1980, they lack immunity against monkeypox virus. Three subjects self-reported prior smallpox vaccination, but the authenticity remains uncertain due to absence of documentation.

All cases reported in this study were mild, and the general population may have a relatively low tendency to seek medical treatment once exposed, and the incubation period of monkeypox was relatively long. These factors, to some extent, increase the risk of epidemic spread.

Rash or skin lesions and fever were the main clinical manifestations of monkeypox cases, similar to those reported in studies conducted in Europe and the United States (18, 27). In the clinical study of monkeypox cases in Nigeria from 2017 to 2018, 65.7% of the patients presented with a rash, and 34.3% with fever as the initial symptom (28), but in another study of monkeypox cases in Congo and Portugal, fever was the main initial symptom (29, 30). In our study, the most frequently reported initial symptom of monkeypox infection was lesions, which is different from the literature studies on symptoms such as fever and headache that occur prior to the rash (24, 30).

Human-to-human or secondary transmission was considered to mainly occur through respiratory droplets during direct and prolonged face-to-face contact, by direct contact with body fluids of an infected person, by contact of mucosa or nonintact skin with open rash lesions, or by contact with contaminated objects (24). Sexual transmission was first proposed in the 2017 outbreak in Nigeria, happening in both male and female patients, and was regarded as possible in the current outbreak, as it mainly affects men who self-identify as having sex with men and have reported recent sex with new or multiple partners (31). Nevertheless, since the current outbreak in 2022, skin lesions around the genitals had become more prevalent. In the context of our study, half of the skin lesions were located in the genital area, and 41.21% were in the limbs region. These findings imply that these areas should be given particular attention during the visit process.

Existing epidemiological investigations have demonstrated that the MSM population exhibits a strong sense of privacy protection and sensitivity, which is related to public opinion pressure (32). In this study, the sexual partners of cases were primarily met through internet-based platforms. However, participants were either unable to provide specific details about these partners or declined to disclose such information. These factors considerably affect the identification of the source of infection and close contacts, which is not conducive to epidemic control. In general, monkeypox surveillance in the region needs to be strengthened.

In summary, this study provides a comprehensive description of the epidemiology and clinical characteristics of the monkeypox cases reported in Zhejiang Province in 2023, which helps us to better understand monkeypox and has certain significance for its early detection and diagnosis. It is imperative to enhance monkeypox surveillance and epidemic information reporting, and to increase public awareness and training for critical demographics and medical staff. However, our research also has limitations. Firstly, most of the monkeypox cases were detected through active clinical visits, and some mild cases and asymptomatic patients may not be screened. Secondly, we only studied the early clinical characteristics of monkeypox cases and lacked data on the entire disease course. Thirdly, some patients may refuse to answer or cannot guarantee the authenticity of the information, which may lead to information bias.

## Data Availability

The original contributions presented in the study are included in the article/supplementary material, further inquiries can be directed to the corresponding authors.
